# Obturator Foramen Bypass With Fluoroscopic Guidance for Recurrent Femoral Prosthetic Bypass Occlusions: A Case Report

**DOI:** 10.7759/cureus.102824

**Published:** 2026-02-02

**Authors:** Hiroto Yasumura, Kenichi Arata, Ryo Imada, Hideyuki Satozono, Koichiro Shimoishi, Yoshihiro Fukumoto, Goichi Yotsumoto, Yuki Ogata, Tomoyuki Matsuba, Yoshiharu Soga

**Affiliations:** 1 Cardiovascular Surgery, Kagoshima City Hospital, Kagoshima, JPN; 2 Cardiovascular Surgery, Graduate School of Medical and Dental Sciences, Kagoshima University, Kagoshima, JPN

**Keywords:** cfa, clti, fluoroscopy, obturator foramen bypass, prosthetic bypass occlusions, zeego

## Abstract

Repeated and additional prosthetic bypass to the femoral artery causes femoral crowding and adhesion and increases the risk of bypass infection. The femoral crowding may cause the stenosis of native and prosthetic bypass during hip flexion. An obturator foramen bypass is a bypass from a particular branch of the abdominal aorta to the lower limb artery to circumvent groin infection. This procedure is particularly effective in treating infected femoral prosthetic bypasses and mycotic femoral artery aneurysms; however, the bypass is routed through a narrow obturator foramen, which requires advanced technical skills.

A 57-year-old Japanese man with a history of a coronary stent occlusion and a terminal aorta-left common femoral artery (CFA) prosthetic bypass occlusion underwent a right external iliac artery (EIA)-left superficial femoral artery (SFA) crossover prosthetic bypass. At the age of 61 years, the patient presented with pain in the left first toe with purulent discharge and noticed that the toe turned purple when his left hip joint was flexed during soaking in the bathtub. Contrast-enhanced computed tomography revealed occlusion of the right EIA-left SFA crossover prosthetic bypass and left narrow and shaggy CFA and SFA. Compression of the left CFA between the two occluded prosthetic bypasses and the caput femoris during hip flexion was considered to limit the blood flow to the lower limb, leading to chronic limb-threatening ischemia (CLTI). Those multiple femoral prosthetic bypasses had resulted in left femoral crowding, raising concerns that a new bypass to the left CFA might be compromised by the dynamic femoral compression during hip flexion. Therefore, an alternative bypass route was necessary. We successfully performed a 25-cm thrombectomy of the left femoral artery, followed by a right common iliac artery (CIA)-left SFA crossover prosthetic bypass through the left obturator foramen under live fluoroscopic guidance using Artis zeego (Siemens Healthineers, Erlangen, Germany). The postoperative course was uneventful. The graft remained patent over 12 months.

Obturator foramen bypass may be considered an option not only in avoiding inguinal infections but also in addressing femoral crowding and compression caused by hip flexion. Penetrating the obturator foramen should be performed at the center or lower position. Preoperative vascular assessment of the foramen ovale on CT is also important. The procedure can be safely performed under live fluoroscopic guidance.

## Introduction

The common femoral artery (CFA) is located as shallow as 2-4 cm below the skin of the inguinal site. Repeated and additional prosthetic bypass to the femoral artery causes femoral crowding and adhesion and increases the risk of bypass infection. Prosthetic graft infections occur in 3.8% of bypass procedures involving the femoral artery during a mean follow-up period of 27 months [1].

Hip flexion can cause a CFA stenosis of more than 50% in patients with non-atheromatous disease [2]. The femoral crowding due to repeated prosthetic bypass procedures may cause the stenosis of native and prosthetic bypass during hip flexion.

An obturator foramen bypass is usually a bypass from a particular branch of the abdominal aorta to the lower limb artery to circumvent groin infection. This procedure is particularly effective in treating infected femoral prosthetic bypasses [3] and mycotic femoral artery aneurysms [4]; however, the bypass is routed through a narrow obturator foramen, which requires advanced technical skills. We describe a rare case of obturator foramen bypass for chronic limb-threatening ischemia (CLTI) induced by femoral crowding due to multiple prosthetic bypasses and hip flexion, using fluoroscopic guidance.

## Case presentation

A 57-year-old Japanese man with hyperglycemia and hypertension underwent emergency percutaneous coronary intervention (PCI) for acute myocardial infarction of the right coronary artery. He had a 37-year smoking history of 20-40 cigarettes per day, and his father had a history of stroke. After the PCI, he was prescribed aspirin (100 mg) and prasugrel (3.75 mg); however, the drug-eluting stent was occluded four days later, necessitating another emergency PCI. During the hospitalization, the patient complained of three-year-longstanding intermittent claudication after walking 100 meters (Fontaine classification Stage Ⅱ b) [5]. Fontaine classification is staged from I to Ⅳ. Stage I is asymptomatic, meaning there are no symptoms despite the presence of arterial disease. Stage II is characterized by intermittent claudication and is divided into two subgroups: Stage IIa, where walking distance is greater than 200 meters (mild claudication), and Stage IIb, where walking distance is 200 meters or less (moderate to severe claudication). Stage III involves ischemic rest pain, indicating more advanced circulatory compromise. Stage IV represents the most severe stage and includes ulceration or gangrene, consistent with CLTI.

**Figure 1:**
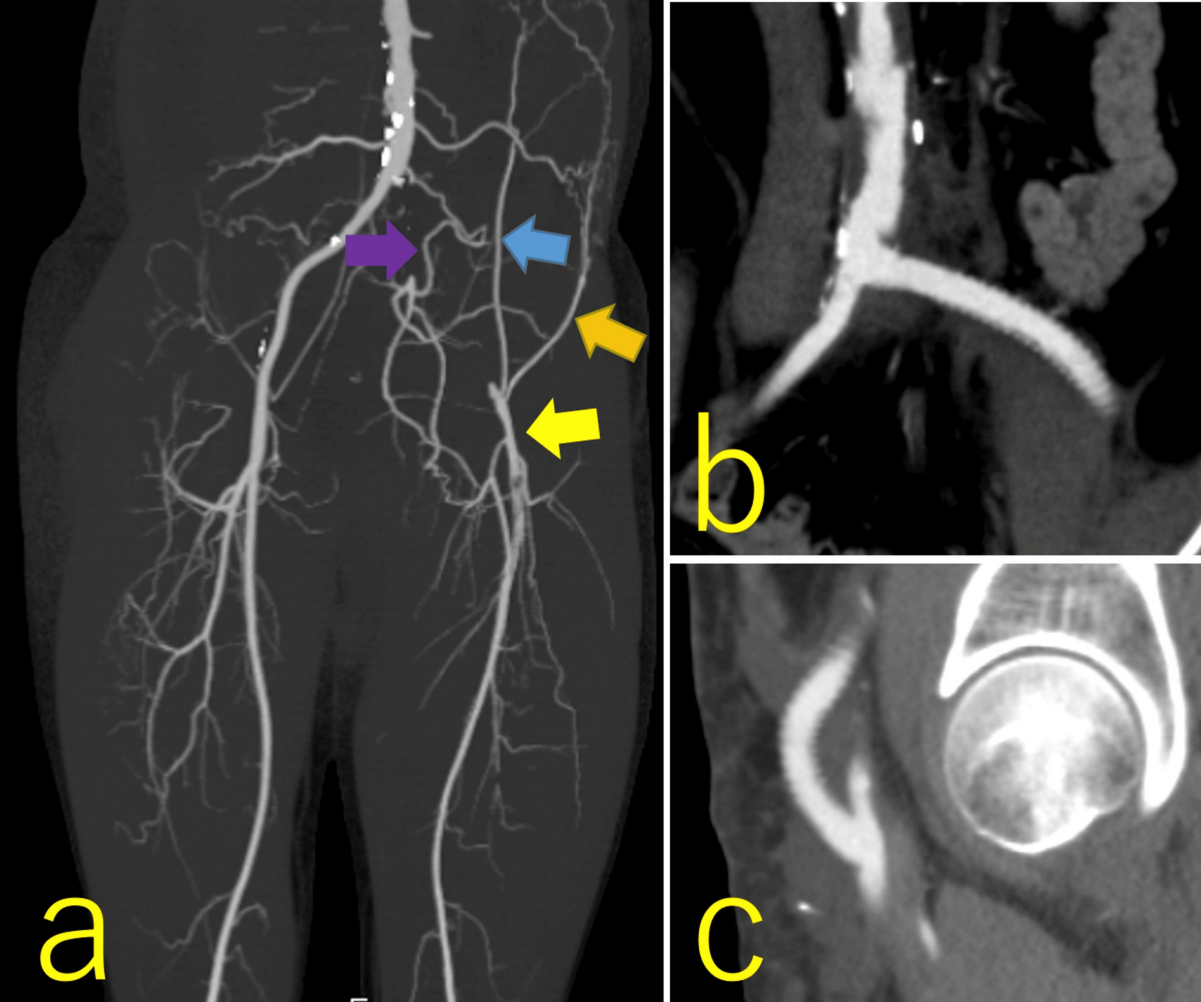
Pre- and postoperative computed tomography (CT) findings of the prosthetic bypass between the terminal aorta and left common femoral artery (CFA) a: occlusion from the left common iliac artery to the CFA, and developed collateral arteries, including the left lumbar artery (purple arrow), inferior epigastric artery (blue arrow), and circumflex artery (orange arrow), which drained into the left CFA (yellow arrow); b: proximal anastomosis site of the prosthetic bypass between the terminal aorta and left CFA; c: distal anastomosis site of the prosthetic bypass between the terminal aorta and left CFA.

A contrast-enhanced CT scan revealed chronic total occlusion from the left common iliac artery (CIA) to CFA (Trans-Atlantic Inter-Society Consensus Class Ⅱ: D lesion). The CT also showed that collateral arteries that had developed on the left, including the lumbar arteries, inferior epigastric artery, and iliac circumflex artery, drained into the left CFA (Figure 1a). His left ankle brachial index (ABI: ankle systolic blood pressure/highest brachial systolic blood pressure [6]) was 0.52. There were no specific findings on blood thrombotic factors. Trousseau’s syndrome was suspected, and examinations of tumor markers, positron emission tomography, CT, and endoscopy were performed. However, no malignancies were observed.

**Figure 2:**
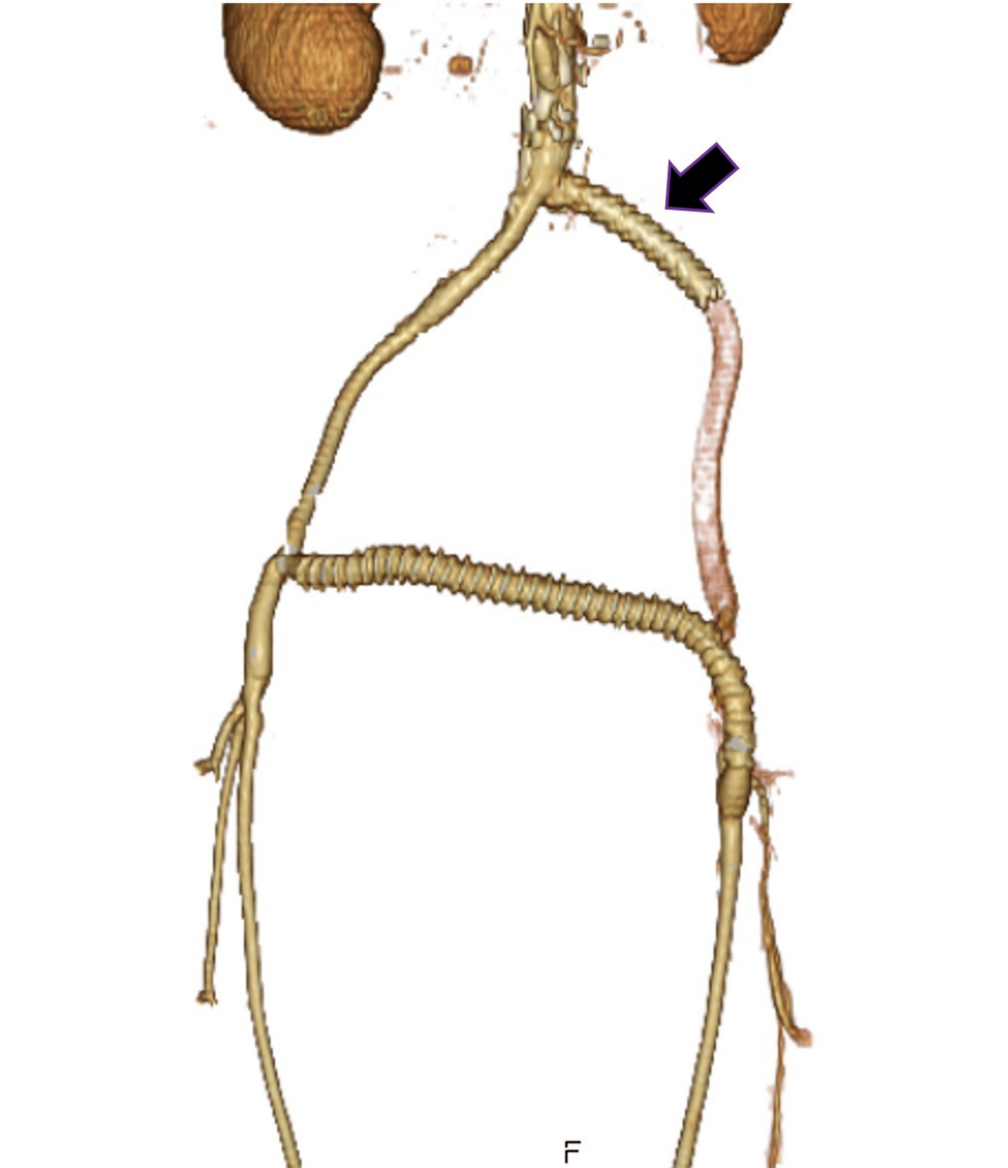
Postoperative CT finding of an emergent crossover prosthetic bypass An emergent crossover prosthetic bypass between the right EIA and left SFA was performed through the prevesical space. The black arrow is the previous 8 mm Epic Vascular Stent. EIA: external iliac artery; SFA: superficial femoral artery

Four months after discharge, the patient (168.4 cm, 68.4 kg) was referred to our hospital for revascularization. The therapeutic lesion was as long as 32 cm, and the surgical procedure was preferable to endovascular therapy (EVT). Thrombectomy was performed from the left CFA to the superficial femoral artery (SFA), and prosthetic bypass grafting was performed between the terminal aorta (Figure 1b) and left CFA (Figure 1c) using an 8 mm Advanta VXT (Getinge, Gothenburg, Sweden) through the retroperitoneal space. On postoperative day (POD) 1, clopidogrel sulfate (75 mg), aspirin, and prasugrel were prescribed. The patient was discharged on POD 10. However, the CFA prosthetic bypass was occluded within two months postoperatively. We performed thrombectomy for the occluded bypass and EVT for the proximal anastomotic mild stenosis using an 8 mm Epic Vascular Stent (Boston Scientific, Massachusetts, USA); however, two hours after the procedure, the patient complained of sudden numbness and pain in the left leg, and echography revealed that the bypass was occluded again. Therefore, we performed emergent crossover prosthetic bypass grafting between the right external iliac artery (EIA) and left SFA using an 8 mm Fusion (Getinge, Gothenburg, Sweden) through the prevesical space (Figure 2).

Postoperative blood tests for thrombophilia indicated negative heparin-induced thrombocytopenia antibody and low plasminogen activity (43% (reference value, 71-128%)), which was considered complicated serological and vascular coagulopathy due to smoking and hyperglycemia. Hence, edoxaban tosilate hydrate (60 mg) was added to the patient’s medication regimen. The patient was discharged on POD 11.

Three years after the crossover bypass, at the age of 61 years, the patient experienced pain in the left first toe with purulent discharge (Fontaine classification Stage Ⅲ) and noticed that the toe turned purple when his left hip joint was flexed during soaking in the bathtub. The patient visited our hospital again. We could not palpate the left dorsalis pedis or the posterior tibial arteries. The left ABI of the patient could not be measured (Table 1).

**Table 1:** Preoperative, intraoperative, and postoperative data of obturator foramen bypass After obturator foramen bypass, symptoms and ABI were improved, and the bypass remained patent for 12 months. ABI: ankle brachial index; CFA: common femoral artery; SFA: superficial femoral artery; TTFM: transit time flow measurement

Data		Preoperative findings	Intraoperative findings	Postoperative findings		
				One week later	6 months later	12 months later
Age (years)		61			62	
Limb symptoms		Pain in the left first toe, which turned purple during soaking in the bathtub		None	None	None
Blood flow measurement using the Doppler method	Left CFA	D-2, 49 cm/sec				
	Left SFA	D-3		50 cm/sec	44 cm/sec	40 cm/sec
	Left Pop. A	D-3, 13 cm/sec		D-2, 43 cm/sec		
	Obturator foramen bypass		220 mL/min PI 0.9 (TTFM)	Patent 50-119 cm/sec	Patent 32-160 cm/sec	Patent 39-150 cm/sec
ABI	Left	Unmeasurable		0.84	0.78	0.84
	Right	1.17		1.1	1.12	1.1

**Figure 3:**
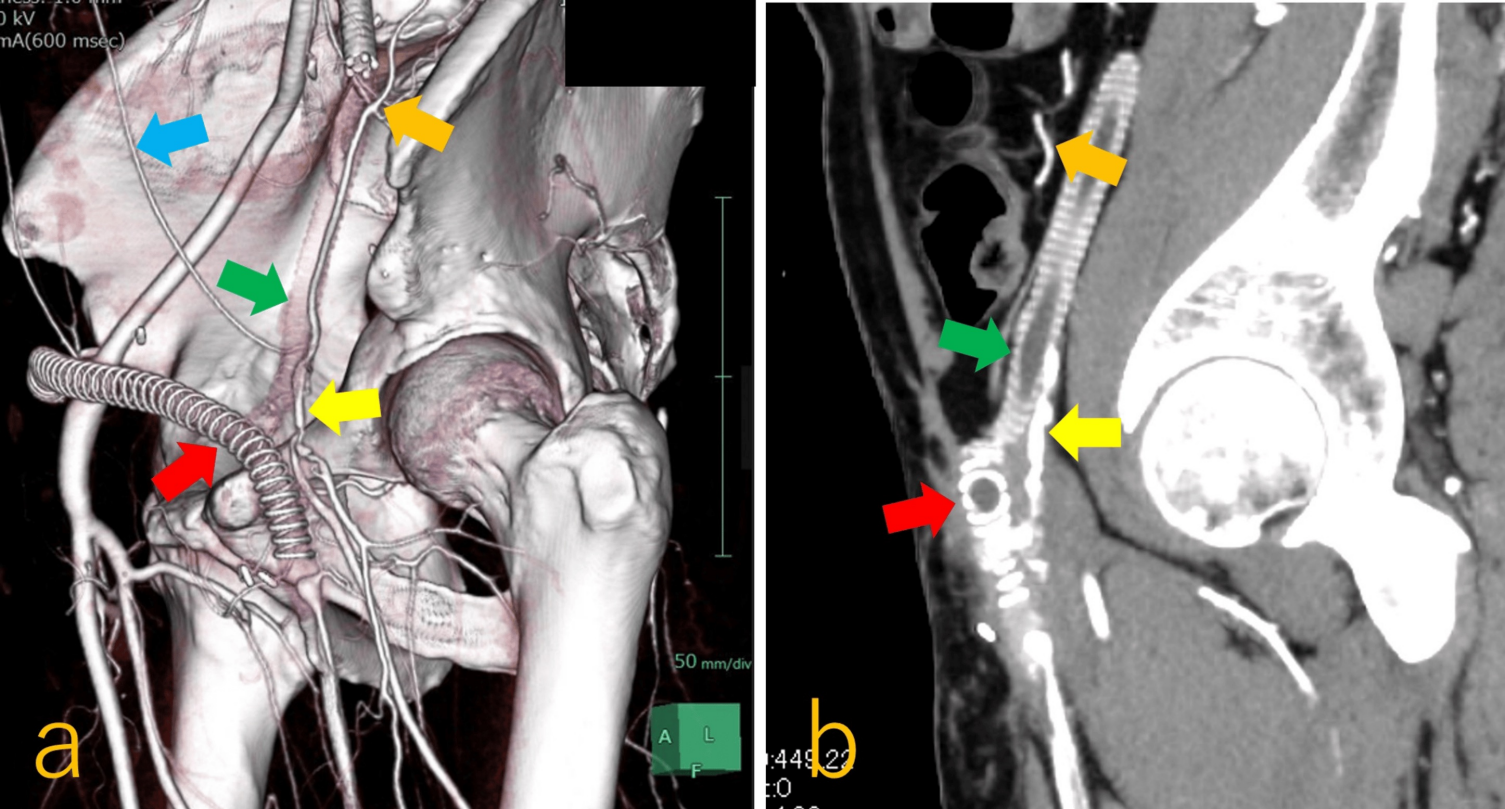
Preoperative CT finding of obturator foramen crossover bypass grafting a: three-dimensional CT showing the two occluded prosthetic bypasses: the green arrow indicates a terminal aorta–left CFA bypass, whose distal anastomosis was detached, and the red arrow indicates a right EIA–left SFA crossover bypass, the blue arrow marks the inferior epigastric artery, the orange arrow indicates the iliac circumflex artery, and the yellow arrow identifies the CFA; b: the left CFA (yellow arrow) and SFA were narrow and shaggy, the CFA was suspected to be compressed between the two prosthetic bypasses (green and red arrows), the caput femoris when the hip joint was flexed, and the orange arrow indicates the iliac circumflex artery. EIA: external iliac artery; SFA: superficial femoral artery; CFA: common femoral artery

Contrast-enhanced CT revealed that the crossover bypass was occluded, the left CFA and SFA appeared narrow and shaggy (Figures 3a, 3b), and the deep femoral artery (DFA) was patent. He was diagnosed with CLTI. Compression of the left CFA between the two occluded prosthetic bypasses and the caput femoris during hip flexion was considered to limit the blood flow to the lower limb. We were concerned that a new subcutaneous femoral bypass to the left CFA would be compromised by the dynamic femoral compression due to graft crowding during hip flexion. Therefore, we opted for obturator foramen crossover bypass grafting between the right CIA and left SFA. Preoperative blood examination revealed no specific abnormality.

**Figure 4:**
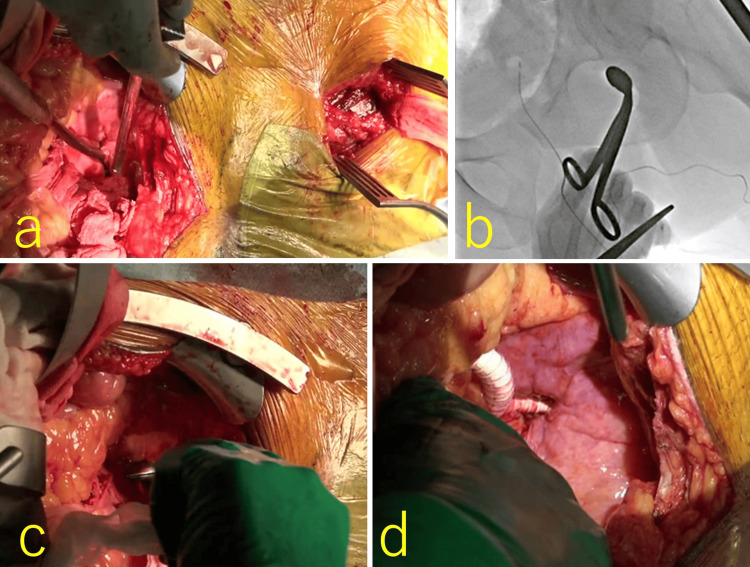
Intraoperative findings of obturator foramen crossover bypass grafting and thrombectomy a: the tunneller could not communicate between the transabdominal retroperitoneal pathway and the post-adductor longus muscle pathway; therefore, we added a left inguinal incision; b: we could not pull through the obturator foramen and used live fluoroscopic guidance; c: fluoroscopic guidance enabled us to obtain safe and successful tunneling through the obturator foramen from the inguinal site; d: we anastomosed an 8-mm PROPATEN graft to the right common iliac artery and tunneled it through the left obturator foramen via the retroperitoneal pathway.

We performed an abdominal median incision, incised the retroperitoneum from the terminal aortic bifurcation to the outside of the sigmoid colon, and tunneled the sigmoid colon mesenterium. After identifying the bilateral obturator foramina lateral to the bladder, we incised the left middle thigh, released the Hunter canal, and exposed the narrow SFA. However, the tunneler could not communicate between the trans-abdominal retroperitoneal and post-adductor longus muscle pathways. Therefore, a left inguinal incision was made (Figure 4a) to obtain the post-sartorius muscle pathway. Although we could not initially pull through the obturator foramen, fluoroscopic guidance using Artis zeego (Siemens Healthineers, Erlangen, Germany) (Figure 4b) enabled us to obtain safe and successful tunneling through the obturator foramen from the inguinal site (Figure 4c). We then anastomosed an 8 mm PROPATEN (Gore, New Jersey, USA) 80 cm to the right CIA and tunneled it through the left obturator foramen via the retroperitoneal pathway (Figure 4d).

**Figure 5:**
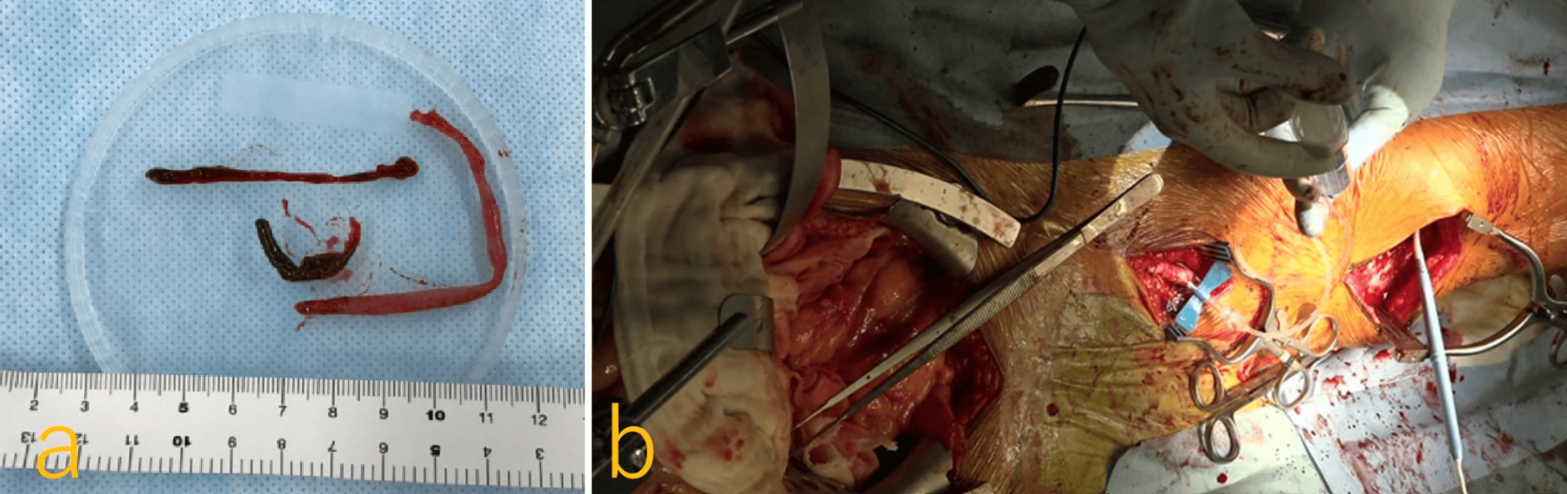
Intraoperative findings of obturator foramen crossover bypass grafting and thrombectomy a: 25-centimeter massive thrombus was removed from the left CFA to SFA; b: prostaglandin was injected from the prosthetic bypass graft, and blood flow measured at the distal SFA was 220 mL/min with a PI of 0.9. CFA: common femoral artery; SFA: superficial femoral artery

Additionally, we performed a thrombectomy for a 25-cm massive red thrombus that extended from the left CFA to the SFA (Figure 5a) to ensure as much blood flow as possible to the DFA and anastomosed the PROPATEN graft to the left SFA. Transit time flow measurements at the distal SFA (Figure 5b) indicated a flow rate of 220 mL/min (SFA reference value, 226.2 ± 82.1 mL/min [7], 93.6 ± 14.1 cm/sec [8]) and a pulsatile index of 0.9 (Table 1).

**Figure 6:**
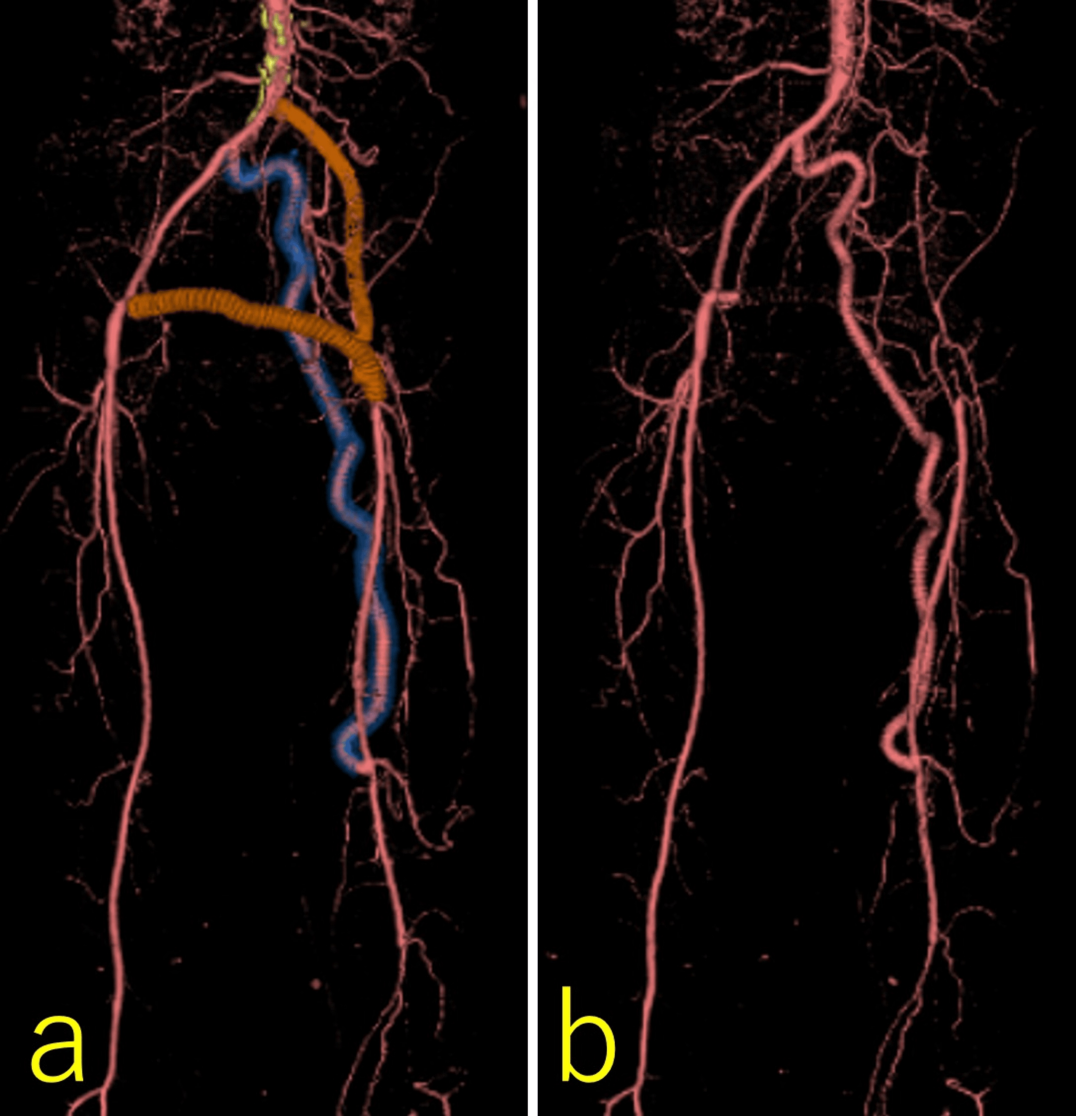
Postoperative findings of obturator foramen crossover bypass grafting and thrombectomy a: contrast-enhanced three-dimensional CT showing the previously implanted grafts, demonstrating good patency of the obturator foramen crossover bypass; b: contrast-enhanced three-dimensional CT without visualization of the previously implanted grafts.

The patient’s postoperative course was uneventful, and contrast-enhanced CT on POD 6 confirmed good patency of the obturator foramen crossover bypass (Figure 6). His left ABI improved to 0.84. He was discharged on POD 15. The graft surveillance was performed every six months by ultrasonography. The graft remained patent over 12 months postoperatively (Table 1).

## Discussion

Shaw and Baue first reported the obturator foramen bypass in 1963 [9]. Although this technique is considered "extra-anatomical," it offers a shorter and straighter route compared to the subcutaneous pathway, which contributes to longer-term graft patency. Sautner reported that the primary patency rate of obturator foramen prosthetic bypass was 75% at one year and 54% at five years [10]. Similarly, Masaki noted patency rates of 69% at three years and 43% at five years for prosthetic bypasses [11]. Although an autologous vein graft provides longer patency, the saphenous veins were as narrow as 1 mm at the knees, and they were not suitable as a bypass graft in our case.

Obturator foramen bypass performed to avoid physical compression is rare, and this is the first such report to our knowledge. We also considered a redo femoral-femoral crossover bypass and left axillofemoral bypass; however, these options require a bypass to route the subcutaneous pathway and cross the hip joint. As such, they would be more susceptible to surgical site infections and compression during hip flexion, which is why they were discontinued. Surgical removal of the previously implanted grafts was not a therapeutic consideration due to the strong adhesion of the bypass graft, either.

Obturator foramen bypass requires surgeons to work in a confined space within the pelvic floor. Therefore, care must be taken not to injure the bladder, ureter, obturator artery, or vein during tunneling. Magishi reported a case in which an obturator foramen crossover prosthetic bypass penetrated the bladder [12]. In our case, we were able to identify both obturator foramina on the pelvic floor; however, we could not clearly locate the obturator artery and vein. Passing the graft through the narrow obturator foramen is technically challenging [13]. We initially advanced a tunneler bluntly, but the tip veered off course beneath the ischium and strayed under the adductor magnus, which is why the transabdominal and inguinal pathways did not communicate. Live fluoroscopic guidance using Artis zeego from multiple angles is helpful for confirming the correct passage of the tunnel through the obturator foramen [13]. The most important consideration when passing through the obturator foramen is bleeding due to vascular injury. The obturator artery and vein route at the upper medial position of the obturator foramen [14], and penetrating the obturator foramen should be performed at the center or lower position. Preoperative vascular assessment of the foramen ovale on CT is also important.

## Conclusions

Obturator foramen bypass may be considered an option not only in avoiding inguinal infections but also in addressing femoral crowding and compression caused by hip flexion. Penetrating the obturator foramen should be performed at the center or lower position. Preoperative vascular assessment of the foramen ovale on CT is also important. The procedure can be safely performed under live fluoroscopic guidance.
